# Combined SERS-Raman screening of HER2-overexpressing or silenced breast cancer cell lines

**DOI:** 10.1186/s12951-024-02600-7

**Published:** 2024-06-20

**Authors:** Sara Spaziani, Alessandro Esposito, Giovannina Barisciano, Giuseppe Quero, Satheeshkumar Elumalai, Manuela Leo, Vittorio Colantuoni, Maria Mangini, Marco Pisco, Lina Sabatino, Anna Chiara De Luca, Andrea Cusano

**Affiliations:** 1 Optoelectronic Division-Engineering Department, University of Sannio, Benevento, 82100 Italy; 2 Centro Regionale Information Communication Technology (CeRICT Scrl), Benevento, 82100 Italy; 3 Institute for Experimental Endocrinology and Oncology G. Salvatore, IEOS, second unit, Via P. Castellino 111, Naples, 80131 Italy; 4 Department of Sciences and Technologies, University of Sannio, Benevento, 82100 Italy; 5 Biosciences and Territory Department, University of Molise, Pesche, 86090 Italy

## Abstract

**Background:**

Breast cancer (BC) is a heterogeneous neoplasm characterized by several subtypes. One of the most aggressive with high metastasis rates presents overexpression of the human epidermal growth factor receptor 2 (HER2). A quantitative evaluation of HER2 levels is essential for a correct diagnosis, selection of the most appropriate therapeutic strategy and monitoring the response to therapy.

**Results:**

In this paper, we propose the synergistic use of SERS and Raman technologies for the identification of HER2 expressing cells and its accurate assessment. To this end, we selected SKBR3 and MDA-MB-468 breast cancer cell lines, which have the highest and lowest HER2 expression, respectively, and MCF10A, a non-tumorigenic cell line from normal breast epithelium for comparison. The combined approach provides a quantitative estimate of HER2 expression and visualization of its distribution on the membrane at single cell level, clearly identifying cancer cells. Moreover, it provides a more comprehensive picture of the investigated cells disclosing a metabolic signature represented by an elevated content of proteins and aromatic amino acids. We further support these data by silencing the *HER2* gene in SKBR3 cells, using the RNA interference technology, generating stable clones further analysed with the same combined methodology. Significant changes in HER2 expression are detected at single cell level before and after HER2 silencing and the HER2 status correlates with variations of fatty acids and downstream signalling molecule contents in the context of the general metabolic rewiring occurring in cancer cells. Specifically, HER2 silencing does reduce the growth ability but not the lipid metabolism that, instead, increases, suggesting that higher fatty acids biosynthesis and metabolism can occur independently of the proliferating potential tied to HER2 overexpression.

**Conclusions:**

Our results clearly demonstrate the efficacy of the combined SERS and Raman approach to definitely pose a correct diagnosis, further supported by the data obtained by the *HER2* gene silencing. Furthermore, they pave the way to a new approach to monitor the efficacy of pharmacologic treatments with the aim to tailor personalized therapies and optimize patients’ outcome.

**Supplementary Information:**

The online version contains supplementary material available at 10.1186/s12951-024-02600-7.

## Background

Breast cancer is the most frequent malignancy among women in developed countries and the leading cause of cancer-related death [1]. BC is a heterogeneous pathology and cancer-bearing patients with apparently similar clinical-pathological characteristics may face different clinical courses. On the basis of cytological and molecular biomarkers, invasive BCs are classified into four molecular subtypes with the following gene expression profiles [2]:

1. Luminal A: neoplasms with marked expression of estrogen (ER) and progesterone (PR) hormone receptors (ER + and/or PR+).
2. Luminal B: neoplasms with a high risk of recurrence, although expressing hormone receptors (ER + and/or PR + and HER2+);
3. Triple negative (TNBC): neoplasms characterized by the absence of hormone receptors and HER2 expression;
4. HER2 enriched (HER2+): characterized by the overexpression of the HER2 receptor (ER-, PR- HER2+).

This latter subtype accounts for 20–30% of all invasive BCs and it is characterized by a remarkably high expression of HER2, an orphan tyrosine kinase receptor involved in proliferation signalling, varying from 500.000 to more than 2 million molecules per cell compared to the 25.000 to 185.000 present in normal epithelial cells or non-amplified tumours [3].

HER2 is an important clinical biomarker, as its overexpression or amplification is associated with unrestricted tumour growth, greater aggressiveness, metastasising rate and resistance to chemotherapy influencing patient management [4, 5]. Quantifying HER2 positivity is crucial for a correct and early diagnosis; to this end, several methods have been standardized and routinely used such as: (i) immunohistochemistry (IHC), (ii) fluorescence in situ hybridization (FISH) and (iii) chromogenic in-situ hybridization (CISH) [6]. IHC recognizes and quantifies the HER2 protein in a tissue sample, while in FISH a fluorescent probe hybridizes with *HER2* genomic sequences allowing the identification of the gene copy number per cell. CISH is a diagnostic method that combines the chromogenic signal detection, as in IHC, with in-situ hybridization. Despite their wide use, these techniques have several limitations. They require tissue biopsies and cannot be performed on single cells, often are not sensitive enough to detect HER2 at clinically relevant levels, are time-consuming, have a limited dynamic range strongly influenced by the individual assessment of physicians, which frequently leads to disagreements between pathologists [6]. It has been reported that about 20% of HER2 positive diagnoses, made with the available standard methods, appear to be inaccurate [7].

Additionally, HER2 overexpression has been exploited as a potential therapeutic target for the clinical management of HER2 + BC patients, as in the case of monoclonal antibodies (trastuzumab, TZ, and more recent derivatives) directed against HER2 [8]. Alternatively, HER2 silencing via RNA interference (RNAi) has been proposed as treatment of HER2 + tumor cells [9–14]. In this case, a double-stranded RNA recognizes the target mRNAs inducing either sequence-specific cleavage and degradation or a translational block. The recurrence rate in BC treated with HER2 therapy has been associated with drug resistance; however, this effect has not been completely clarified, due to the lack of rapid and accurate techniques that can easily quantify an appropriate dynamic HER2 range in patients [15]. Indeed, monitoring the response to HER2-targeted therapies and understanding the biochemical characteristics of single tumors could strongly contribute to the development of targeted and personalized therapeutic approaches by improving the efficacy of existing treatments and optimizing their outcomes.

Methods and technologies for a precise HER2 quantification in patients are of fundamental relevance for proper BC diagnosis, for guiding physicians in elaborating the more appropriate therapeutic strategy and predicting the response to the selected treatment. Specifically, a method able to assess at single cell level the exact HER2 concentration and the associated cellular metabolic changes would provide a great benefit for studies on cell lines in vitro and for the diagnosis of HER2 + BC in vivo.

Recently, Raman microscopy has shown promising results as an analytical tool in precision medicine, especially in the classification of cancer subtypes and in evaluating their biochemical composition at subcellular compartment level [16]. Raman spectroscopy relies on inelastic light scattering, which is directly related to the molecular vibrational energy specific for each molecular bond. This technique offers several advantages such as no sample preparation, fast, label-free and multiplexing identification of chemical species coupled with high spatial and lateral resolution in a confocal setup. In this way, the specific metabolic signature of a cancer cell or tissue can be detected, as reported in several ex vivo and in vitro studies on various cancers including thyroid, ovarian and leukemias [16–18].

Correctly identifying Raman bands among hundreds of spectral features has become a common practice with the advent of Machine Learning (ML) techniques that can extract valuable information from large datasets with an enormous number of independent variables. Typical approaches are unsupervised algorithms such as Principal Component Analysis (PCA) able to count on the similarities of distances between different objects. In alternative, supervised algorithms such as Linear Discriminant Analysis (LDA) can be applied for specific transformations to find relationships between the explanatory and independent variables with already known classes. The most recent application of supervised ML was on seven different BC cell lines reported by Zhang et al. who achieved 97% and 92% accuracy in discriminating among normal and cancer cell lines, and among all the cell lines, respectively [19, 20].

Among the Raman based-techniques, surface-enhanced Raman spectroscopy (SERS) has emerged as a powerful tool that demonstrates its potential to detect and quantify membrane biomarkers, including HER2 levels, through a nanoparticle-based approach [21, 22]. SERS, characterized by high sensitivity and molecular specificity, represents a paradigm shift in the field of vibrational spectroscopy. In fact, unlike conventional Raman spectroscopy, SERS exploits the plasmonic properties of noble metal nanoparticles, particularly gold and silver, to amplify the Raman scattering signals of analytes adsorbed on their surfaces [23]. The amplification factor can reach values of 6–10 orders of magnitude, allowing detection of even trace amounts of analytes with unprecedented precision [24].

By functionalizing nanoparticles with ligands, antibodies, or aptamers it would be possible to selectively target and capture HER2. Indeed, the binding event amplifies the HER2 Raman signal, making it easily detectable even in the smallest amounts. This combination of selectivity, sensitivity, and molecular specificity renders SERS a breakthrough technique for the detection and quantification of membrane biomarkers, particularly HER2 in BC [25, 26].

Therefore, while Artificial Intelligence (AI)-assisted Raman spectroscopy can provide efficient cell discrimination and identify the specific metabolic signature of cancer cells, SERS analysis can provide a detailed visualization of the distribution of biomarkers on the cell surface. The information obtained by Raman spectroscopy and SERS complements each other and enables a synergistic enrichment of the clinical relevance of the collected data. Nonetheless, despite the numerous works carried out, either on classification of BC cell lines using Raman spectroscopy [19, 20, 27–29] or HER2 detection using SERS strategies [26, 30], a combined SERS-Raman approach for BC cells analysis and HER2 expression assessment has not been investigated so far.

In the present study, we propose a SERS-Raman screening of some representative BC cell lines both for quantitative identification of HER2 levels and for correlating its expression and membrane exposure with the specific metabolic features associated with malignant transformation. The proposed global approach has the potential to improve diagnostic capabilities enabling a more nuanced and accurate assessment of the metabolic signature associated with this type of cancer cells. Furthermore, we applied the same SERS-Raman approach to cell clones in which we preliminarily stably silenced the *HER2* gene using the RNA interference technology [10, 12]. This approach permits to investigate the metabolic changes associated with HER2 expression and increases the accuracy and reliability of the Raman analysis itself, as all observed signals can be confidently attributed to the presence or absence of HER2 minimizing background interference from the expression of the endogenous receptor. Overall, the association of the SERS-Raman spectroscopy with the RNA interference technology provides valuable insights into the biology of HER2 + BC cells and, in particular, in a condition that mimics the effect of HER2-silencing targeted therapies [10, 12], a theme that is novel and not explored so far. The acquired knowledge can contribute to ongoing research efforts aimed at developing new and more effective therapeutic interventions in HER2 + BC and to assess the efficacy of HER2-targeted therapies.

## Methods

### Cell culture

The human breast cancer-derived cell lines SKBR3 (ATCC catalogue number HTB-30), BT-474 (ATCC number HTB-20), MCF7 (ATCC number HTB-22), MDB-MB468 (ATCC number HTB-132), MDA-MB-231 (ATCC number HTB-26), along with MCF10A (ATCC number CRL-10,317), representative of the normal breast epithelium, were purchased from the American Type Culture Collection (ATCC, Rockville, MD, USA) and cultured at 37 °C under 5% CO_2_ in DMEM or RPMI (Life Technologies-Thermo Fisher Scientific, Waltham, MA, USA) supplemented with 10% v/v fetal bovine serum (Life Technologies-Thermo Fisher Scientific, Waltham, MA, USA), 2 mM L-glutamine, and 100 U mL-1 penicillin/streptomycin (15,140,122 Gibco™ Thermo Fisher Scientific, Waltham, MA, USA). BT474 cells were grown in a medium containing 10% insulin, while MCF10A cells in a DMEM/F12 medium supplemented with L-glutamine and Pen/Strep (standard concentrations), 5% horse serum, 0.5 µg/mL hydrocortisone (stock solution prepared dissolving hydrocortisone in ethanol), 100 ng/mL cholera toxin, 10 µg/mL insulin, 20 ng/mL EGF (human). Cells were checked regularly to exclude any contamination with mycoplasma.

For Raman and SERS experiments, all cell samples were plated on CaF_2_ Raman grade slides, fixed with 3% paraformaldehyde and stored in PBS at 4 °C before use. Specifically, for SERS imaging, all samples were passaged in 1% BSA in 2 mM BB for 1 h to reduce nonspecific binding between the functionalized nanoparticles and the CaF_2_ slide. Raman measurements were performed after washing the cells with a fresh PBS buffer solution.

### Western blot analysis

Protein extracts were analysed as previously reported [31]. Antibodies to p-HER2 (Y1248 # 2247), HER2 (#3250), p-AKT (S473) (#9271), AKT (#9272) were from Cell Signaling Technology (Beverly, MA USA); α-Tubulin (T5168) was from Sigma-Aldrich Co. (Merck KGaA, St. Louis, MO, USA). Anti-mouse or anti-rabbit antibodies conjugated with horseradish peroxidase, were used as secondary antibodies. Bands were detected by the Clarity western ECL Substrate (#1,705,061, Bio-Rad, Hercules, CA, USA), using the ChemiDoc XRS Apparatus (Bio-Rad, Hercules, CA, USA). The intensity of the bands was evaluated by ImageLab software (Bio-Rad, Hercules, CA, USA).

The same cell lines were additionally tested for their ability to release the HER2 extracellular domain The conditioned media were harvested from cells cultured for 48 h, concentrated with AmiconUltra® (Merck Millipore, St. Louis MO, USA) to load the same amounts of proteins (50 µg/sample) for all samples to the polyacrylamide gel. Soluble HER2 was detected with an antibody against the N-terminal part of the receptor (#4290 Cell Signaling Technology, Inc., Beverly, MA USA). The amount of protein released into the medium was calculated by loading the same protein content for each sample and normalizing the values to those obtained by Ponceau S staining of the membrane.

### HER2 silencing

The SKBR3 cells were plated in 6 wells in normal culture medium and incubated for 12 h in controlled temperature and CO_2_ conditions. Subsequently, the chosen vector, HER2 (ERBB2) Human shRNA Plasmid Kit (Locus ID 2064, OriGene code TG320342) and the corresponding negative control vector (scramble shRNA control) were transfected with the Lipofectamine™ reagent. The vectors used carry the gene coding for the resistance to the antibiotic puromycin, the gene for the Green Fluorescence Protein (GFP) used as a reporter of transfection efficiency, and a transcriptional unit in which the U6 promoter was fused to a DNA fragment that synthesises a short hairpin RNA specifically directed against *HER2* mRNA. Forty-eight hours after transfection, cells were split (1:10) and placed in fresh medium, under selection with an adequate concentration of puromycin (3 µg/mL, previously determined with a resistance curve using non-transfected cells). Only those cells that had acquired the plasmid and thus had acquired resistance to the antibiotic were selected and subjected to further amplification and testing for HER2 expression.

### Flow cytometry analysis for HER2 exposure on the plasma membrane

SKBR3, BT474, MCF7, MDA-MB-468, MDA-MB-231 along with MCF10A cells (3 × 10^5^) were plated overnight under standard conditions; the next day cells were harvested, washed three times with PBS pH 7.4 containing Ca^2+^/Mg^2+^ and fixed with 3% PFA at RT for 10 min. After washing, cells were stained with a primary PE-CY7 conjugated anti-HER2 antibody (324,414 Biolegend, San Diego, CA, USA). Finally, cells (20.000 events for each cell line) were evaluated by flow cytometry and the results analysed using FACSuite software v.1.0.5.3841 (BD Biosciences, Franklin Lakes, NJ, USA).

Statistical analyses were performed using GraphPad Prism 6 (GraphPad Software Inc, San Diego, CA, USA). Data are expressed as mean ± SEM of independent experiments performed at least in duplicate.

### SERS nanoparticles preparation and characterization

AuNps are functionalized sequentially with the Raman reporter and TZ antibody. Briefly, 40 nm gold colloidal NPs were combined with 50 mM borate buffer (BB) to adjust the pH to 8.5; this suspension was then mixed with a 1 mM 4-mercapto-benzoic acid (4-MBA) solution in methanol. This solution was mixed by inversion and then incubated at 4˚C for 90 min. To activate the -COOH groups, freshly prepared 1-ethyl-3-(3-dimethylamino-propyl)carbodiimide (EDC − 0.4 M) and N-Hydroxysuccinimide (NHS − 0.1 M) in 0.1 M 2-(N-morpholino)ethanesulfonic acid (MES) pH 4.8 were added to a suspension of nanoparticles and stirred continuously for 5 min. Excess of EDC and NHS were removed by centrifugation and the particles were re-suspended in MES buffer. AuNps were then immunosensitsed by adding 100 µg/mL solution of TZ antibody in MES buffer and allowed to react at 4 °C for 90 min. After washing three times by centrifugation to remove non-conjugated antibody, a 10% (w/v) bovine serum albumin (BSA) solution in BB pH 8.5 was added, which promoted colloidal stability and blocked unreacted binding sites. After centrifugation, TZ-AuNPs were re-suspended in 2 mM BB, supplemented with 1% (w/v) BSA, and stored at 4 °C until use. The TZ-AuNps were incubated with the cells for 90 min. at room temperature. After three washes with 2 mM BB and 1% (w/v) BSA.

The TZ-loaded gold nanoparticles (TZ-AuNps) were characterised by dynamic light scattering to determine whether the functionalization protocol was successful and to ensure that the particles were still monodisperse (Additional file, Table S1). Due to the covalent conjugation of 4-MBA and TZ antibody and BSA coverage, the TZ-AuNps were approximately three times larger (Figure S1). The functionalization procedure and the presence of the Raman reporter, TZ antibody, and BSA on the AuNps were both confirmed by the decrease in the negative surface charge from − 39.7 (± 1.02) mV of the bare AuNps to -28.8 (± 0.93) mV. In addition, we also measured the polydispersity index (PDI), a parameter that is crucial in nanoparticle applications because it provides information on the uniformity of the sample after surface conjugation and aggregation state. The PDI is essentially a measure of the heterogeneity or breadth of the particle size distribution, and in our case the PDI value was 0.25 ± 0.03 confirming the stability of the TZ-AuNps preparation and the monodisperse distribution [32].

TZ was conjugated to the AuNps using an amine coupling reaction. Therefore, it is not possible to determine the orientation of the antibody and consequently to be sure that the recognition efficiency remained unchanged compared to the unconjugated antibody. To assess that the TZ immobilized onto the nanoparticles could still detect HER2, we performed ELISA assays and compared the results with those pertaining to free TZ. Both TZ-AuNps and free TZ are able to detect HER2 in a dose-response manner, over the entire concentration range tested (1–10–100 ng/ml). This demonstrates the high specificity and selectivity of the TZ antibody after the immobilization on the NPs (Additional file, Figure S2) and also confirms a high conjugation rate of TZ.

Furthermore, we investigated the presence and stability of 4-MBA on the TZ-AuNps by characterizing the intensity and reproducibility of the SERS signal. Figure S3 in Additional file shows the average SERS spectra of 4-MBA recorded from the TZ-AuNps deposited on the quartz slide which are characterized by two intense bands at 1080 and 1580 cm^− 1^, corresponding to the vibrations of the aromatic ring of the 4-MBA molecule [33]. The calculated enhancement factor EF value for 4-MBA TZ-AuNps was approximately 2.3 × 10^6^ (± 0.2) [34]. Therefore, the TZ-AuNps probe can specifically recognize the HER2 protein and enhance the 4-MBA Raman signal by up to six orders of magnitude. In order to have a negative control, we also functionalized nanoparticles with the Raman reporter and a specific and selective antibody for a different protein [34]. The incubation of the functionalized nanoparticles with SKBR3 (HER2+) cells was carried out in the same conditions, demonstrating the absence of aspecific binding.

### Raman and SERS measurements, processing and data analysis

Horiba XploRA INV Raman confocal Microscope equipped with a 532-nm wavelength diode Laser (2 mW on the sample) and a 60x water immersion objective (NA = 1.2) was used for the Raman measurements. The spectra were acquired with a holographic grating of 600 lines/mm, a confocal pinhole of 100 μm and an entrance slit of 300 μm, allowing a resolution of 4 cm^− 1^ and a beam waist of 300 nm. 30 cells for each cell lines were analyzed with at least 9 spectra for each cell, mapping the cytoplasmic cell region. The spectra are collected with an integration time of 60s and cropped in 800–3100 cm^− 1^ spectral region. Data analysis has been performed in R environment. Rubber band algorithm provided with the *HyperSpec* R package has been used for removing from all the spectra the contribution of the water (main broad band at 2500–3500 cm^− 1^) and the sample autofluorescence. It fits the baseline by a smoothing spline through the supporting points, that is subtracted to the Raman spectrum. Finally, the residual baseline has been removed by fitting an Asymmetric least square algorithms (Als), implemented in the R Baseline’s package, which applies iterative second derivative constraints on a weighted regression to fit the baseline. Finally, the pre-processed spectra have been vector normalized.

Classification of BC cell lines was performed by applying two ML techniques: the principal component analysis (PCA) and linear discriminant analysis (LDA), implemented in the two R packages prcom (base R package) [35]. PCA multivariate analysis decomposition was performed via single value decomposition (SVD), to decompose data matrix in three matrixes: loadings, scores, and variances for each component. To evaluate the chemical difference provided by the classification for PCA loadings of the firsts 4 PC, counting for more than 90% of cumulative variances of each component has been plotted with the median ± IQR (interquartile range), and compared with reference bands of vibrational modes of the cell main constituents (Table 1). Hence, to visualize the performance of the PCA classification, scores of PC2-4 have been plotted in a 3-dimensional space. Eventually, the PCs components best explaining the class differences has been evaluated by Wilcoxon non-parametric test and visualized by a box plot of PC scores versus the BC cell lines. LDA analysis has been applied to the spectra with 6 cm^− 1^ resolution, and the model was trained on 50% of the data, and the overall accuracy of the model has been calculated as mean misclassification error over 1000 iterations on a ten-fold random test set of equal class size distribution. LDA model stability has been shown as a scatter plot of number iterations in the test dataset versus misclassification error. To graphically inspect LDA classification performances, for each LDA a scatter plot with latent variables LD1-LD2 has been produced.

SERS maps were acquired using the 785 nm single-mode diode laser (delivering a power of 2 mW on the sample) as the excitation source. Three SERS maps were acquired for each cell line and each map corresponds to several SERS spectra, in the range 600–1700 cm^–1^, depending on the size of the cell. We applied an integration time of 0.5 s and a step size of 1 μm, thus each map took about 5 min. To highlight the presence of Raman bands associated with the Raman reporter (4-MBA), we show the colour maps in terms of the intensity of the bands at 1080 and 1580 cm^− 1^, respectively. To quantify the SERS response, we counted the characteristic spectral peaks of the 4-MBA that result from the background noise and occur at both the 1080 and 1580 cm^− 1^ Raman shifts. In particular, we determined the noise threshold for each SERS map by considering the region of the SERS spectra where no band of interest was present using an appropriate Matlab code (Additional file, Figure S4). These thresholds were calculated using the equation T = mp + 3σp [34], where mp and σp are the mean and standard deviation of the maxima SERS intensity peaks in the range between 890 and 910 cm^− 1^. We obtained similar intensity maps showing only peaks that simultaneously exceed the respective noise thresholds at both Raman shifts (Additional file, Figure S4B). Therefore, to count the “number of pixels” associated with the SERS signal from the Raman reporter in one map, we only considered the “bright” pixels for both intensity maps in Figure S4C of Additional file [34]. The total pixel number of the two SERS maps was used as an indicator of the SERS intensity response.

## Results

### Selection of the normal and breast cancer cell lines

**Fig. 1:**
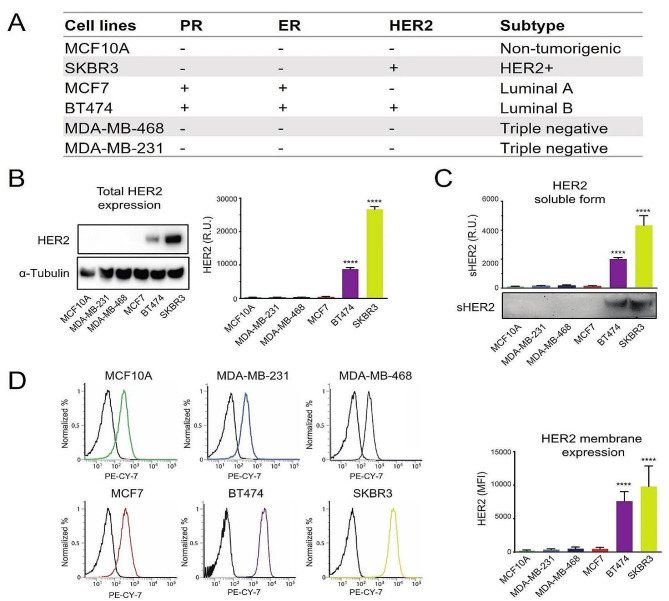
Assessment of HER2 expression in a series of breast cell lines. (**A**) List of the cell lines employed in this study (**B**) Western blot analysis of HER2 expression on extracts from MDA-MB-231, MDA-MB-468, MCF7, BT474 and SKBR3 cells derived from different BCs; MCF10A cells, derived from non-tumorigenic breast epithelium, were used for comparison. α-Tubulin was used to normalise the amounts of proteins loaded in each lane. The histogram reports HER2 total expression as Relative Units (R.U.) compared to MCF10A cells. Statistical significance is considered when **** *p* < 0.0001 (*ANOVA*). (**C**) Western blot analysis of the soluble form of HER2 (sHER2) present in the concentrated media from the same cells as in B). The histogram shows the relative quantification, expressed as Relative Units (R.U.), after normalization to protein loading through Ponceau S staining of the filter. Statistical significance is considered when **** *p* < 0.0001 (*ANOVA*). (**D**) Flow cytometry analysis of HER2 cell surface exposure in the same cell lines as in A). The percentages of cell counts are reported on the y axis, while the Fluorescence Intensity on the x axis. The plots also report the positions of the isotypes as black lines. The histogram shows the relative quantification, expressed as Mean Fluorescence Intensity (MFI). Statistical significance is considered when **** *p* < 0.0001 (*ANOVA*)

We screened a panel of BC derived cell lines for HER2 expression levels by western blot analysis: SKBR3 and BT474 cells, representative of the HER2 + and HER2 + and estrogen receptor+ (ER) subtypes; MCF7, MDA-MB-468, MDA-MB-231 cell lines, representative of the estrogen receptor positive (ER+) and of the triple negative subtypes, respectively; MCF10A cell line from non-tumorigenic breast epithelium was used for comparison (Fig. 1, A). HER2 protein level was elevated in SKBR3 and BT474 cells while all the others displayed very low, if any, expression, similar to the MCF10A cells. α-Tubulin was used as a control to normalize for protein loading; the histogram reports HER2 relative expression referred to this internal control (Fig. 1, B). The same cells were additionally tested for the ability to release the HER2 extracellular domain. A band of about 100 kDa, corresponding to sHER2, could be detected only in the conditioned medium from SKBR3 and BT474 cells, confirming the characteristics of these cell lines (Fig. 1, C). Then, we assessed HER2 exposure on the cell surface by flow cytometry (Fig. 1, D). The receptor was significantly exposed only on the surface of SKBR3 and BT474 cells, while the other cell lines displayed very low levels, similar to the normal MCF10A cell line, as illustrated by the corresponding plots where the isotype peak, typical for each cell line, is also indicated. The histogram reports the quantification of the data expressed as mean fluorescence intensity (MFI) in each cell line.

Starting from these preliminary analyses, we selected the tumor cell lines SKBR3 and MDA-MB-468 as they exhibit the highest and lowest levels of HER2 expression, respectively, and are representative of the most severe prognostic scenarios in BC. The MCF10A cells, derived from non-tumorigenic breast epithelium, were selected for comparison.

### SERS characterization of normal and breast cancer cell lines

We performed SERS experiments on the above indicated cell lines to assess HER2 expression, using as probe gold nanoparticles (40 nm in diameter) functionalized with a Raman reporter and a specific and selective antibody for detecting HER2 as a recognition element. In particular, we functionalized the nanoparticles using 4-MBA, which consists of a thiol group, a benzene ring and a carboxyl group [36, 37]. This molecule plays a dual role as the Raman reporter and conjugating agent: the thiol groups bind to the gold surface and the carboxyl group interacts with the amino groups of the antibody molecules. In this way, the functionalization protocol is simplified and performed directly, without using the core-shell structure to separate the Raman reporter layer from the target molecules, improving the stability and reproducibility of the SERS probe [31, 34, 38]. We selected the Trastuzumab (TZ) antibody as a recognition element because it targets and binds HER2, inhibits the signalling pathways that promote cell growth, helping to restrain the uncontrolled growth of cancer cells [39, 40]. TZ has become a standard of care for HER2 + BC patients and is often administered as part of the adjuvant therapy after surgery to reduce the risk of recurrence [41, 42].

**Fig. 2:**
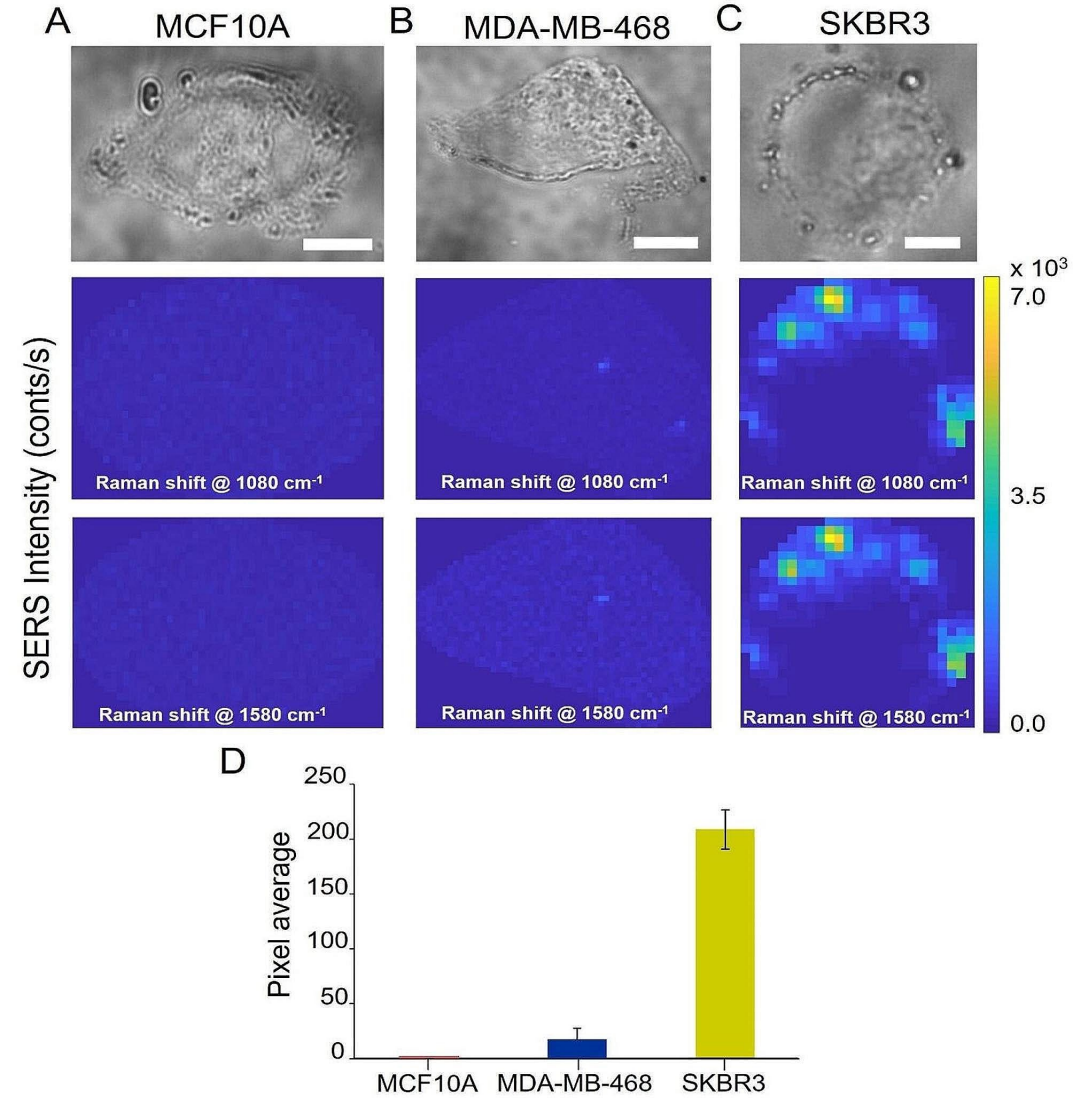
Brightfield imaged and SERS intensity maps of a selected cell for the Raman peaks at 1080 and 1580 cm^− 1^ for the cell lines: (**A**) MCF10A (**B**) MDA-MB-468 (**C**) SKBR3. Scale bar = 10 μm. (**D**) Relative quantification of HER2 biomarker on cell membrane of the analysed cell lines based on the SERS analysis

In order to evaluate the SERS applicability in identifying breast cell lines with different HER2 levels, the TZ-AuNps probe was incubated with three cell types: SKBR3, MDA-MB-486 and MCF10A. Figure 2 shows the brightfield image of a selected cell for each cell line and colour maps of SERS intensity of the bands at 1080 and 1580 cm^− 1^, only showing those pixels whose intensity is higher of the noise threshold simultaneously for both considered bands. The experiment was performed on 10 cells per cell line and repeated 3 times. As expected, SKBR3 cells are characterized by an abundant HER2 receptor on their surface, which the TZ-AuNps recognize and bind to, as illustrated by the pixel average calculated for all cells analyzed for each cell line. In the triple negative MDA-MB-468 cells, HER2 expression is only 8% as compared to SKBR3, while no signal is detected in the non-tumorigenic MCF10A cell line, as HER2 basal expression is extremely low [43]. These data also confirm that the TZ-AuNps do not induce non-specific effects on the cell membrane, but that the binding is the result of the specific and selective recognition of HER2 by the Trastuzumab antibody on the surface of the TZ-AuNps and not to aggregation of the nanoparticles that preserve the same physical characteristics detected by DLS and PDI analysis (see Methods’ section).

It is evident that our approach can clearly distinguish HER2+ (SKBR3) from triple-negative (MDA-MB -468) BC cells and from non-tumorigenic breast epithelial cells (MCF10A). TZ-AuNps are an excellent SERS probe to assess and quantify HER2 directly on the cell membrane, at single cell level, and thus to identify and discriminate cells on the basis of their HER2 expression. However, to assess the biochemical composition of the cells, shed some light on the ongoing tumorigenic mechanisms and correlate with tumour aggressiveness and metastasising potential, additional information is needed.

### Raman characterization of the normal and breast cancer cell lines

We then performed the Raman analysis on the same breast cell lines to correlate the information on the quantitative HER2 expression, obtained via SERS analysis, with a more general metabolic and physio-pathological picture. To this aim, Raman analysis of single cells using the same microscope assisted equipment was performed.

**Fig. 3:**
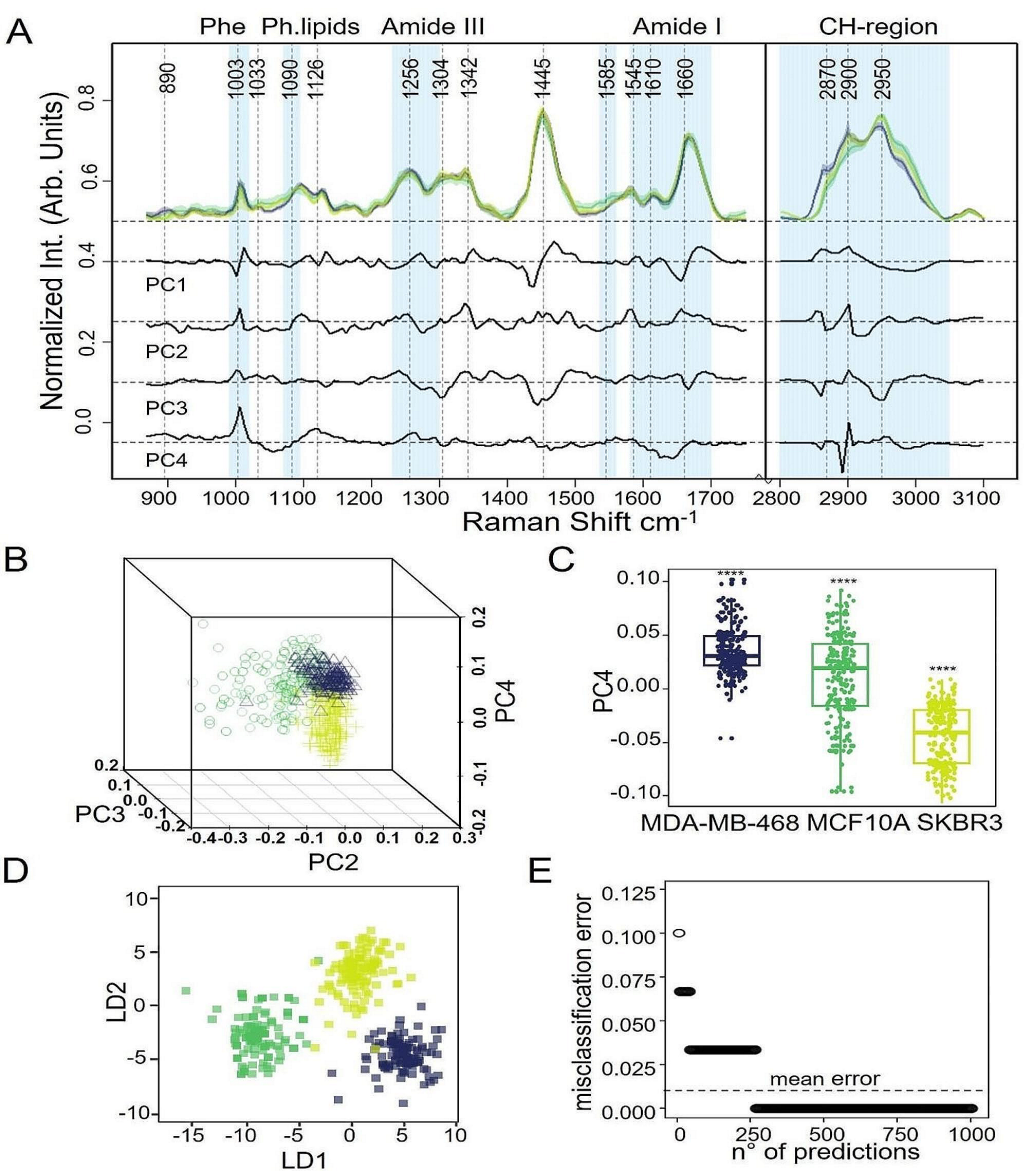
Raman classification of breast derived cell lines expressing different HER2 levels. (**A**) Median + IQR spectra of SKBR3 (lime), MCF10A (green) and MDA-MB-468 (blue), with PCA loading expressing high variance, black lines (y is offset for clarity). Vertical dash lines are single peaks of interest; cyan vertical bands are relevant biological bands in cell classification. (**B**) 3D score plot of PC2-4 scores demonstrating the classification achieved for SKBR3, MCF10A and MDA-MB-468 cells using 3 components. (**C**) Box-plot of the distribution for each cell line of PC4 scores. The asterisks represent Wilcoxon nonparametric test results with *p* < 0.0001 when ****. (**D**-**E**) LDA supervised classification with the separation achieved using the latent variables 1 and 2, and the misclassification rate, respectively

In this frame, the Raman spectra provide data on the chemical fingerprint of a large number of intra-cellular macromolecules, allowing exploiting the biochemical differences among the investigated cell lines. Specifically, we investigated 30 cells for each cell line by acquiring 9 spectra (60s of integration time per spectra) per cell, collecting the signal from the cell cytosol and repeating the experiment three times. The obtained spectra are reported in Fig. 3. The 800–1200 cm^− 1^ spectral range is associated with the DNA backbone, CO/CC stretching in lipids and CN/CC stretching in proteins and with the DNA backbone vibrational modes. The spectral range, located at 1400–1500 cm^− 1^, corresponds to the CH, CH2 and CH3 stretching modes, mainly associated with proteins and lipids, while that at the 1500–1750 cm^− 1^ region corresponds to different modes of lipids (C = C stretching) and proteins (Amide I band, C = 0 stretching). In addition, the CH region between 2700 and 3100 cm^− 1^ is representative of membrane lipids: specifically, fatty acids and phospholipids in the region at 2700–2900 cm^− 1^ while proteins and lipids at 2900–3100 cm^− 1^. A more detailed picture with band designation is reported in Table 1 [19, 44, 45]. To have a clearer representation of the chemical differences among the cell lines and to evaluate them in terms of classification performances, we carried out PCA and LDA analysis associated with ML techniques (Fig. 3). Specifically, for the PCA we selected the first four PCs, responsible for about 90% of total data variance, demonstrating the highest variations among lipid and protein bands, that are commonly identified as cancer related macromolecules [19, 21, 46]. With a focus on lipid and proteins, we identified two main patterns which allow to distinguish normal from cancer cells as well as to correlate them with the HER2 expression levels, as shown in Fig. 3B, C by the PC2 and PC4 loadings differences in the PCA analysis, respectively, and in the PC2-PC3-PC4 3D score plot. The main differences arise at the 870–880 cm^− 1^ band related to C-H stretching in the tryptophan residues and at the 1000–1004 cm^− 1^ band that corresponds to breathing vibrational modes of the phenylalanine ring. Hence, we can suggest a possible BC classification on the basis of the aromatic amino acids’ levels. Consistently, MDA-MB-468 cells with the highest level of aromatic amino acids display a very high tumour invasiveness [47]. In contrast, the Raman bands at 1080–90 cm^− 1^ and 1453 cm^− 1^, corresponding to phospholipids, display higher intensity in HER2 overexpressing SKBR3 cells than other lower HER2-expressing cells. It is tempting to correlate this spectral evidence with enhanced de-novo lipogenesis associated with HER2 signaling [48]. In line, triple negative cells (MDA-468) display a not-so-high level of lipids while, in sharp contrast, a huge increment of aromatic amino acids. This finding also suggests that the balance between the aromatic amino acids and lipids can be proposed as a feature to distinguish the HER2 + enriched phenotype from the triple negative one. Additionally, all tested BC cells can be distinguished from non-tumorigenic MCF10A cells by a generalized increase of protein, lipids and nucleic acids content (1090 cm^− 1^,1342 cm^− 1^, 1582 cm^− 1^,1660 cm^− 1^), as reported by the negative bands in PC2 loadings, in Fig. 3, A. This is in line with the enhanced proliferation signalling and the reprogrammed metabolism typical of tumor cells in general and of BC cells in particular [49, 50].

We performed a supervised linear discriminant analysis (LDA), by using 50% of data as training set and 50% as test set, to investigate these metabolic differences (the spectra from a given cell line were not mixed into the training and test sets). Interestingly, we achieved an overall 100% accuracy in BC cell lines classification, in line with the data reported in the literature [19, 46] and a mean misclassification error in prediction of less than 5%. Similar results can be achieved by considering only one spectrum per cell and validating it on an independent batch of cells (see Figure S.5 and Figure S.6).

**Table 1:** Raman bands’ attribution for normal breast epithelium and cancer BC cells [19, 44, 45]

band	Assignation	
**850–880**	Ring breathing mode of tyrosine and C-C stretch of proline ring, collagen C-C stretching	
**877 − 876**	Tryptophan, proteins. Antisymmetric stretching in phosphatidylcholine, lipids	
**898–902**	C-O-C; C-C skeletal modes	
**935–950**	Proline, C-C skeletal of collagen backbone, polysaccharides including C-O-C skeletal mode	
**986**	stretching C-C or C-O in ribose	
**1002–1004**	Phenylalanine. CH3 rocking coupled with C-C stretching of carotenoids	
**1033**	C-H in-plane-bending mode of phenylalanine	
**1062–1063**	Chain C-C stretch in lipids; C-O and C-N stretch in proteins; O-P-O stretch in DNA and RNA	
**1081–1096**	O-P-O (stretching PO2) symmetric (Phosphate II) of phosphodiesters, C-C stretching lipids, P-O stretching in phospholipids, C-N stretching in nucleic acids	
**1125–1126**	C-C stretching in lipids; C-N stretching in proteins; C-O in glucose	
**1155–1557**	C-C (and C-N) stretching of proteins and carotenoids	
**1174–1176**	C-H in-plane-bending mode of tyrosine and carotenoids	
**1207–1209**	stretching mode in phenylalanine tyrosine and hydroxyproline	
**1254–1259**	Amide III, adenine, cytosine (protein β-sheet)	
**1268–1278**	Amide III (α-helix), collagen	
**1298–1303**	C-H2 twisting of lipids, phospholipids, and fatty acids	
**1310**	C-H3 C-H2 twisting mode of collagen/lipids	
**1317–1318**	CH2 twist and bend (nucleic acids, proteins, lipids)	
**1335–1342**	C-H3 C-H2 wagging mode of collagen, C-H2 twist and bend (nucleic acids, proteins, lipids); C-H3 of Nucleic Acid and C-H2 wagging mode of collagen;	
**1448–1453**	C-H2 bending mode of proteins. C-H2 (overlapping) asymmetric CH3 bending, and CH2 scissoring (associated with elastin, collagen, and phospholipids)	
**1582**	Pyrimidine ring (nucleic acids) and heme protein	
**1606–1620**	aromatic amino acids, C = C stretching mode of tyrosine and tryptophan (p)	
**1627–1700**	Amide I	
**1657–1660**	aromatic amino acid in proteins and C = C olefinic stretch unsaturated fatty acid	
**1730–1750**	C = O symm. Glycans and glycogen	
**2850–2875**	CH2 symmetric stretch of lipids; CH2 asymmetric stretch of lipids + proteins	
**2885–2908**	CH2 asymmetric stretch of lipids and proteins	
**2945–2957**	CH3 asymmetric stretch of proteins; aliphatic and aromatic CH stretching vibrations in nucleic acids	

### Generation and characterization of HER2-silenced SKBR3 cells and their SERS-Raman analysis in parallel with scrambled-controls

**Fig. 4:**
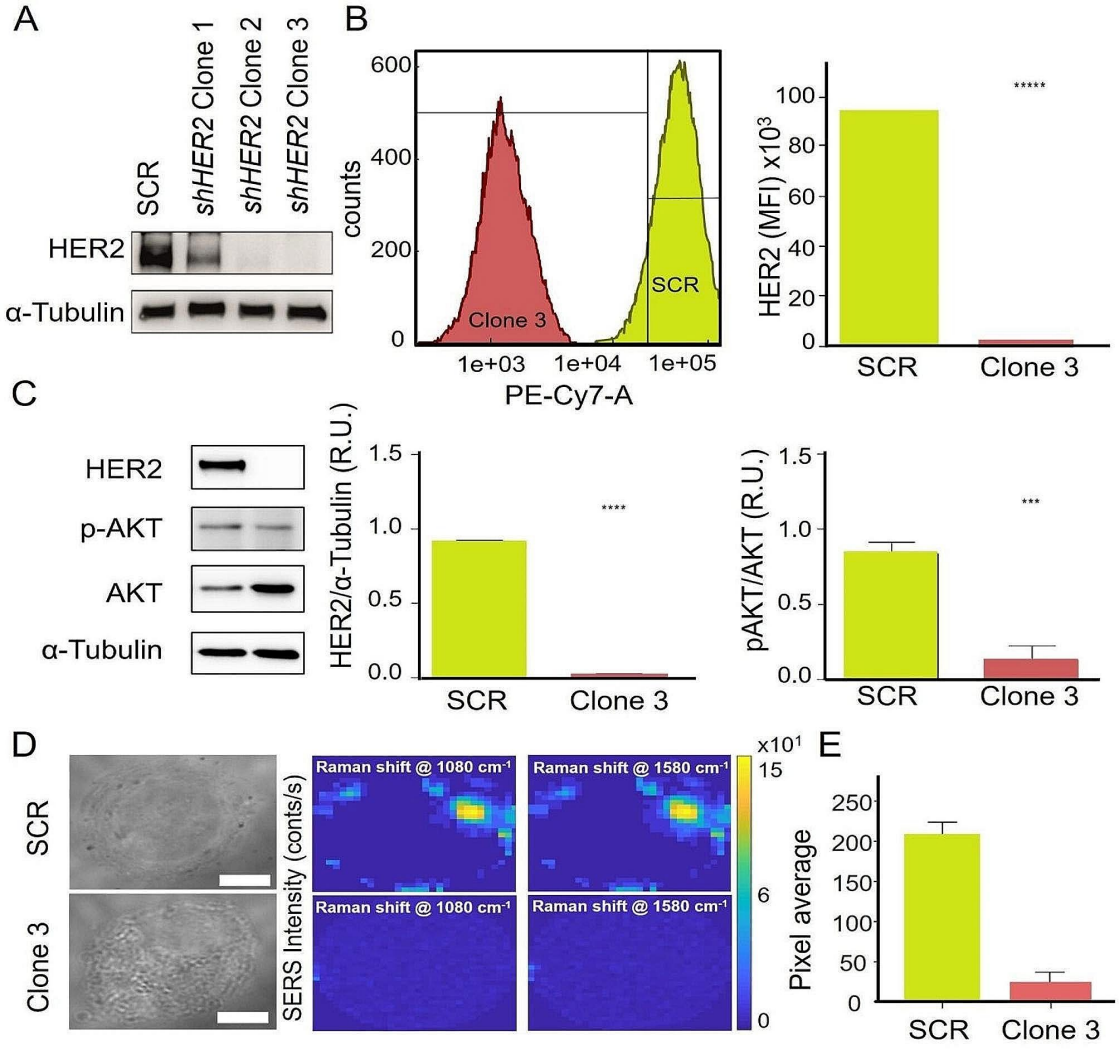
HER2 assessment in scrambled- and HER2-silenced SKBR3 cells. (**A**) Western blot analysis of HER2 on extracts from single clones isolated upon selection of SKBR3 stably transfected with a scrambled control (SCR) or with an shRNA directed against HER2 (HER2 shRNA Clones 1–3). α-Tubulin was used as loading control for each sample. (**B**) Flow cytometry analysis of HER2 cell surface exposure in the scrambled transfected SKBR3 (SCR) or in clone 3 (Clone 3). The cell counts are reported on the y axis, while the Fluorescence Intensity on the x axis. The histogram shows the relative quantification expressed as Mean Fluorescence Intensity (MFI). Statistical significance is considered when **** *p* < 0.0001 (*t*-test). (**C**) Western blot analysis of HER2, AKT and the phosphorylated form levels in extracts from SKBR3 transfected with a scrambled control and from the HER2-silenced clone 3. The first histogram illustrates the quantification of HER2 relative to α-Tubulin and the second the AKT phosphorylation level to total AKT compared to the SCR control. Statistical significance is considered when *** *p* < 0.001 and **** *p* < 0.0001 (*t*-test). (**D**) Brightfield images and SERS intensity maps of a selected cell for the peaks at 1080 and 1580 cm^− 1^ for scrambled- and HER2-silenced SKBR3 cells. Scale bar = 10 μm. (**E**) Relative quantification of HER2 biomarker on cell membrane of the analysed cell lines

In order to simulate the effects of HER2-targeted therapies, we stably silenced the *HER2* gene using the RNA interference technology and applied a combined SERS-Raman approach to analyse the obtained clones. In particular we transfected SKBR3 cells with a plasmid containing a selection marker that confers resistance to the antibiotic puromycin; the gene coding for a Green Fluorescent Protein (GFP) and a transcriptional unit that synthesizes a short hairpin RNA (shRNA) directed against the *HER2* mRNA. The formation of a double stranded molecule between the shRNA and the cognate target mRNA would stimulate the Endonuclease III activity associated with the silencing complex, leading to degradation of the target mRNA and reduced synthesis of the corresponding protein. A plasmid carrying non-specific scrambled sequences was used as negative control (SCR). Transfected cells were selected in a medium containing the antibiotic puromycin and the transfection efficiency assessed by evaluating the expression of GFP under the microscope and HER2 by Western blot analysis. Several independent clones were isolated and tested in parallel with clones from cells transfected with the scrambled control plasmid. Among the selected clones, clone 1 exhibited reduced HER2 levels while clones 2 and 3 showed almost a complete disappearance of the corresponding band. Clone 3 was chosen for further studies as it showed the best silencing (Fig. 4A). The clones from the scrambled control transfected cells displayed HER2 levels similar to the parental cells, as expected. This result indicates that the gene silencing was very efficient. HER2 surface exposure was also tested by flow cytometry (Fig. 4B). Clone 3 displayed an almost undetectable level of HER2 with respect to the SCR transfected cells, with a difference of at least 1.000-fold in terms of mean fluorescence intensity (MFI), corroborating the result of the western blot analysis. We confirmed these results by evaluating in Clone 3 AKT phosphorylation the main intracellular effector of the extracellular signals conveyed *via* HER2. The p-AKT levels significantly diminished with respect to SCR transfected cells (Fig. 4C).

Basically, by using the RNA interference technology, we obtain two cell lines, SCR-SKBR3 and the HER2-silenced counterpart, which represent a unique source as they differ only for HER2 expression. We then applied the SERS technology to differentiate them as shown above. We should emphasize that the SERS approach not only provides a precise HER2 quantification at a single cell basis, but also the specific spatial distribution of HER2 on the cell membrane. In SCR-SKBR3 cells the receptor is highly abundant at the cell boundaries while it is completely absent in HER2-silenced cells, as documented by the remarkably (93%) reduction of the SERS signalling (Fig. 4D).

**Fig. 5:**
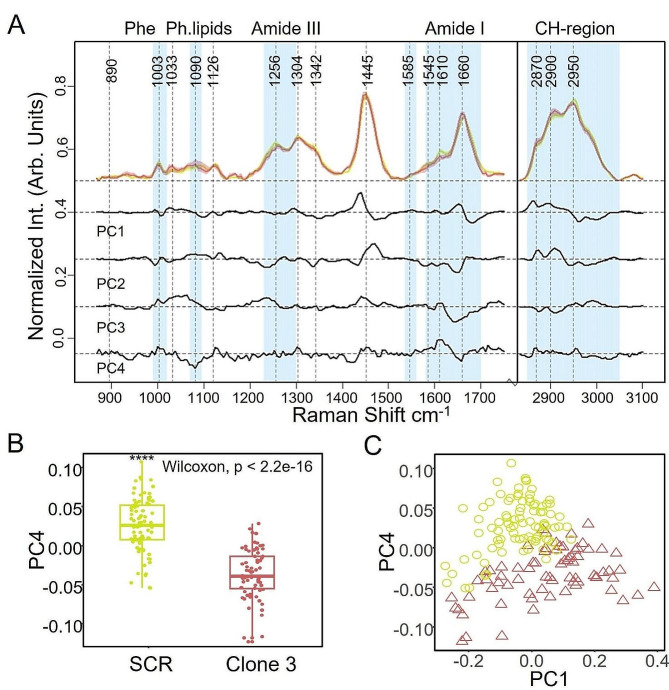
Raman spectra of the scrambled- and HER2-silenced SKBR3 cells (clone 3). (**A**): Median IQR spectra of scrambled (lime) and HER2-shRNA SKBR3 (red) with PCA loadings expressing high variance, as black lines (y is offset for clarity). Vertical dash lines are single peaks of interest; cyan vertical bands are relevant biological bands in cell classification. (**B**) The box plot of distribution of PCA scores for PC4 for the scrambled SKBR3 and silenced counterpart is shown, with a Wilcoxon statistic. (**C**) The score plot of PC1-PC4, evidencing the best spatial separation among scrambled and silenced SKBR3, is shown

We then complemented the SERS data on HER2 expression with those obtained with the Raman spectra, by evaluating the Raman fingerprint of SCR- and HER2-silenced SKBR3 cells by means of investigating 30 cells for each line with 9 spectra (each one with an integration time of 60 s) from different regions of the cytoplasm. The two cell lines displayed only few differences in the spectra, as expected, also in accordance with the lack of morphological changes. The most evident differences were detected in bands located at 1003 cm^− 1^ (phenylalanine), 1081–1096 cm^− 1^ (phosphodiesters, lipids and phospholipids nucleic acids), 1125 cm^− 1^ (C = C in lipids), 1452 cm^− 1^ (proteins and phospholipids associated with proteins), 1585 cm^− 1^ (pyrimidine ring and heme containing proteins) and 1660 cm^− 1^ (aromatic amino acids and unsaturated fatty acids) on the fingerprint region and expressed in the PC4 component with the loading and score plot graphs, respectively, as reported in Fig. 5.

The band at 1003 cm^− 1^, corresponding to the aromatic amino acids content, was reduced in intensity in HER2-silenced SKBR3 (clone 3) cells, in agreement with the concomitant lower intensity of the band at 1610 cm^− 1^, referred to both Amide I band and aromatic amino acids. These data indicate that the HER2-silenced cells have a lower content of aromatic amino acids than the SCR-SKBR3 counterpart, confirming that this selected group of amino acids is heightened in tumour-derived cell lines as a marker of a robust protein synthesis and of the aggressive HER2 + phenotype due to amplification of the HER2 receptor that conveys growth factors stimuli. Specific HER2-enriched Raman fingerprints have not been exhaustively investigated so far and, even in the positive cases, the results are poor or deprived of biophysics characterization. In this context, only a comparative study conducted on the efficacy of lapatinib, a tyrosine kinase inhibitor reports how HER2 expression impacts the Raman fingerprint in triple negative or HER2 + BC cell lines [20, 48].

The broad band located at 1085–1095 cm^− 1^ (phosphodiesters, lipids and phospholipids nucleic acids), representative of the fatty acids chains of phospholipids such as phosphatidylcholine, phosphatidylethanolamine and sphingomyelin [51], showed a slight, not statistically significant difference, suggesting that scrambled and HER2-silenced SKBR3 cells have the same phospholipids content in the cell membranes. An overall spectra analysis in the fingerprinting and -CH region showed that silenced cells display a slightly more intense band at 1080–1090 cm^− 1^, corresponding to phospholipids, while the bands at position 1660 cm^− 1^ and 2850–2900 cm^− 1^, relative to the -CH2 stretching in lipids and to the content of unsaturated fatty acids, are markedly more intense, suggesting that they likely have a higher lipid content and metabolism. All neoplastic cells, as well as BC cells, display an enhanced fatty acids biosynthesis and multiple downstream products content, due to an extensive metabolic reprogramming [52, 53]. This is particularly relevant in cells of the HER2 + subtype, as confirmed by the data reported here and in line with several published RS studies focused on the possible categorization of BC cell lines and in vivo tumor subtypes on the basis of a different biochemical composition. A correlation of BC malignancy with a different saturated/unsaturated lipid composition has been also suggested by Sierra and co-workers, in agreement with the altered lipid metabolism found in cancer progression [54].

Interestingly and quite unexpectedly, HER2-silenced cells exhibit an increase of the lipid content, so that the proliferation potential, related to HER2 overexpression, appears to be dissociated from the metabolic changes, especially of the lipid counterpart. Our finding suggests that the lipid content and fatty acids’ synthesis are regulated through pathways likely independent from HER2 expression, as they persist or even increase upon HER2 silencing. Whether this link is direct or indirect needs to be clarified. Currently, our Raman analysis underscores a lipid metabolism rewiring no longer tied to HER2 expression and to an aggressive behaviour.

## Discussion

In this study, we propose for the first time the combined use of the SERS and Raman technologies to test their performance to identify and quantitate HER2 expression in two selected BC cell lines, SKBR3 and MDA-MB-468, exhibiting the highest and lowest levels of HER2 expression, respectively, together with MCF10A, a non-tumorigenic cell line from the normal breast epithelium. The quantification of HER2 levels in BC patients is of fundamental relevance to a proper diagnosis, to the selection of the most appropriate therapeutic strategy as well as to follow-up the response to therapy. We report that SKBR3 cells display the highest SERS signal, while the triple negative MDA-MB-468 cells a very low signal, corresponding only to 8% of the SKBR3 value; no signal is detected in the non-tumorigenic cell line with an extremely low, if any, HER2 expression. The SERS analysis was carried out by using gold nanoparticles functionalized with 4-MBA, as the Raman reporter, and with TZ, as HER2-specific antibody. These functionalized nanoparticles act as an ultrasensitive and specific SERS nanoprobe, providing an amplification of the Raman signal up to 6 orders of magnitude. Thus, the SERS strategy with TZ-AuNps can clearly recognize and bind HER2 even when expressed at very low levels. The choice of TZ as recognition element offers a double advantage, as it drives the specific binding to the membrane biomarker HER2 and is a standard of care for HER2 + BC patients because it is often administered as part of the adjuvant therapy after surgery to reduce the risk of recurrence [41, 42]. The functionalized nanoparticles act as an ultrasensitive and specific SERS nanoprobe, providing an amplification of the Raman signal up to 6 orders of magnitude and information on HER2 membrane distribution at single cell level. Thus, the SERS strategy with TZ-AuNps can clearly recognize and bind HER2 even when expressed at very low levels, as confirmed in the *HER2* gene stably silenced SKBR3 cells, in which the TZ-AuNPs probe recognizes a 93% difference with respect to the scrambled-transfected counterpart, in line with other biochemical data. These latter results indicate the potential of the association of SERS technology with the gene silencing approach for its use in vivo to monitor the efficacy of HER2-targeted therapies and to follow-up treated patients.

Paralleling SERS analysis, we provide for the first-time evidence that the Raman technology can correlate HER2 expression levels with the cellular metabolic profile. Specifically, the Raman bands corresponding to the aromatic amino acids content show the highest intensity in the triple negative MDA-MB-468 cells which exhibit a highly proliferative potential in line with the notion that tumours with a more aggressive behaviour have an enhanced protein content necessary to meet the higher energy requirements. Bands of lower intensity are detected in SKBR3 cells, representative of the HER2 + BC subtype, consistent with over-expression of the HER2 receptor and with a less aggressive phenotype. A further diminution of band intensity is detected in HER2-silenced SKBR3 cells, in line with a reduced activation of the AKT signalling. The correlation of HER2 levels and aromatic amino acids accumulation with the tumour phenotype appears very solid and could be the basis for translating these results into the analysis of patients’ samples and setting-up an effective analytical device for a rapid diagnosis and intraoperative cancer classification.

Interestingly, the Raman experiments provide hints on the cellular lipidic component. In particular, the Raman phospholipids bands at 1080–1090 cm^− 1^ (lipids, phospholipids and nucleic acids) and at 1453 cm^− 1^ (proteins and phospholipids) exhibit the highest value in HER2 + cells compared to the triple negative and non-tumorigenic cell lines. As mentioned, BC cells exhibit an enhanced synthesis and content of fatty acids and lipid signalling molecules that sustain their proliferation potential. The present data support that cells of the HER2 + BC subtype have the highest level of lipid content, as previously reported [52, 53]. Thus, it is tempting to speculate that this spectral evidence reflects the more robust enhancement of the de-novo lipogenesis associated with or mediated by the amplification of the HER2 signalling.

The finding that HER2*-*silenced SKBR3 cells exhibit a heightened amount of total lipids is quite unexpected, as we hypothesized that this value should be strictly related to HER2 expression and hence to the proliferation potential. We thus suggest that the HER2 + phenotype alone is not stringent enough to modulate the lipid metabolism, that can be related instead to a more synergistic physiological phenomenon in which other factors play a role as, for instance, other ERBB family members (i.e. HER3) involved in lipogenesis and proliferation [55].

The result of HER2-silenced cells underscores the potential of the RNA interference technology as a tool to modulate expression and study gene function in diverse biological processes and explore its possible use in the clinical practice by targeting mRNAs involved in diseases’ pathogenesis. Indeed, small interfering RNAs (siRNAs) have been exploited as therapeutics and some of them approved by the US and European Authorities (FDA and EMA) for selected human diseases [56, 57]. Several siRNA drugs targeting mRNAs or miRNAs with crucial roles in the occurrence of several pathologies, including cancer, involving diverse organs have been developed and tested in vitro, in animal models and in preliminary clinical trials [56–58]. HER2-targeting siRNAs either alone or fused to different aptamers have also been employed with encouraging results in mice models of breast and gastric cancers or in combination with classical chemotherapeutics to increase tumour responsiveness to treatment, especially in conditions of acquired drug resistance [11, 13, 14]. Restoring some physiological functions, as shown here in SKBR3 cells, can be a further therapeutic application of this technology.

In conclusion, the study reported here shows, for the first time, the potential of associating gene silencing with the combined SERS-Raman technology to correctly classify breast cancers, discriminate the various subtypes at single cell level with high specificity and selectivity, and characterize general and distinctive metabolic changes. The overall reliable and consistent results represent the basis to translate the procedure into the clinical practice by analysing patients’ biopsies or tumour tissue specimens to support a correct diagnosis or follow the response to therapy. The practical impact of the SERS-Raman results would be even more relevant if extended to tumours of different origin expressing diverse markers in which the efficacy of selected siRNAs could be tested either alone or in combination with other anticancer drugs to enhance the effects.

Nowdays, SERS-based devices are flexible, hand-held Raman systems commercially available, with the potential to be compact and easily carried in comparison to most of flow cytometers. This consideration opens new avenues for reliable point-of-care applications where a smaller, more portable device is preferred.

### Electronic supplementary material

Below is the link to the electronic supplementary material.

Supplementary Material 1

Supplementary Material 2

## Data Availability

We encourage fellow scholars and the public to use, cite, and share data from this submission in compliance with copyright law, relevant laws and regulations. If you have any questions or requests regarding the use of the data, please contact corresponding authors by email (pisco@unisannio.it; sabat@unisannio.it; annachiara.deluca@cnr.it).
