# Supplementary material for: Combined SERS-Raman screening of HER2-overexpressing or silenced breast cancer cell lines
Source: J Nanobiotechnology. 2024 Jun 20;22:350. doi: 10.1186/s12951-024-02600-7 (PMC11188264; doi:10.1186/s12951-024-02600-7)
Supplement: Supplementary file 1 — Supplementary Material 1 [file 12951_2024_2600_MOESM1_ESM.pdf]

Supplementary Information

Combined SERS-Raman screening of HER2-overexpressing or silenced breast cancer cell lines

Sara Spaziani[§,1,2], Alessandro Esposito[§,3], Giovannina Barisciano[§,4], Giuseppe Quero[5], Satheeshkumar Elumalai[3], Manuela Leo[4], Vittorio Colantuoni[4], Maria Mangini[3], Marco Pisco[1,2,*], Lina Sabatino[4,*], Anna Chiara De Luca[3,*,+], Andrea Cusano[1,2+.]

[1] Optoelectronic Division-Engineering Department, University of Sannio, 82100, Benevento, Italy

[2] Centro Regionale Information Communication Technology (CeRICT Scrl), 82100, Benevento, Italy

[3] Institute for Experimental Endocrinology and Oncology G. Salvatore, IEOS, second unit, Via P. Castellino 111, 80131, Naples, Italy.

[4] Department of Sciences and Technologies, University of Sannio, 82100, Benevento, Italy

[5] Biosciences and Territory Department, University of Molise, 86090 Pesche, Italy

[§]co-first authors

[+]co-last authors

[*]corresponding: pisco@unisannio.it; sabat@unisannio.it; annachiara.deluca@cnr.it

Material and reagent

Gold nanoparticles are from BBI Solutions (Salisbury, United Kingdom). N-Hydroxysuccinimide (NHS), 1-ethyl-3-(3-dimethylamino-propyl)carbodiimide (EDC), phosphate-buffered saline (PBS), borate buffer (BB), bovine serum albumin (BSA), 4-mercaptobenzoic acid, and 2-(N-morpholino)ethanesulfonic acid (MES) were from Merck-Millipore (Milan, Italy). Trastuzumab (Herceptin, Genentech, San Francisco, USA) was obtained in versions and formulations approved by the European Medicines Agency and commercially available. Prior to use, the antibody was purified by dialysis.

TZ-AuNps characterization by dynamic light scattering

To evaluate the functionalization protocol and to verify the stability of TZ-AuNps, the hydrodynamic radius, polydisperse index (PDI) and surface charge (Z pot) were measured by Dynamic Light Scattering using a Zetasizer Ultra instrument (Malvern Instruments Ltd., England) equipped with a 633 nm laser (scattering angle 173°). The dimensions, surface charge, and aggregation state of the TZ-AuNps were first characterized by DLS and compared to naked AuNps. Dynamic light scattering (DLS) analyzes evaluated the size of the gold nanoparticles (~40 nm) (Figure S1) before surface modification, their stability (PDI of 0.13), and a strong negative charge (Table S1). Following the functionalisation protocol, which involved covalent binding of the Raman reporter and antibody and passivation of the remaining gold moieties, the particles appear to have a diameter of 122 nm and a surface charge of -28.8 mV, while maintaining a reasonable PDI (0.25) (Table S1), suggesting monodisperse TZ-AuNps particles (Figure S1).

Table S1 Size by DLS with Polydispersity Index (PDI) values and Zeta Potential (Z pot) data for AuNps.

Sample	Size (d nm)	PDI	Z pot (mV)
40 nm naked	41.26 ± 0.05	0.13 ± 0,01	-39.7 ± 1.02
Post functionalization	122.01 ± 1.82	0.25 ± 0.03	-28.8 ± 0.93

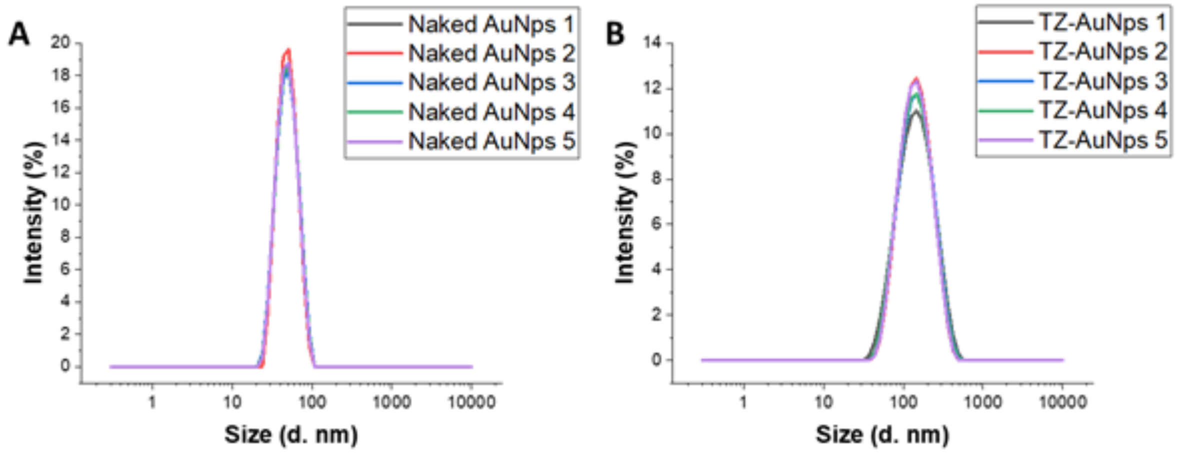

Figure S1 Particle size was determined using DLS. In particular, five measurements of the Z-size of A) Naked and B) TZ-AuNps.

TZ-AuNps recognition efficiency of HER2

TZ-AuNps were characterized by Enzyme Linked ImmunoSorbent Assay (ELISA). All steps were performed at 37°C. For dose–response binding assays, HER2 was loaded at three concentrations (0

– 1 - 10 - 100 ng/mL) onto microtiter ELISA plates in triplicate wells. After a passivation step, the TZ and TZ-AuNps were incubated for 1 hour. Then, it was incubated a HRP-conjugated secondary antibody for 1 hour each well was incubated with QuantaRed™ Enhanced Chemifluorescent HRP Substrate kit according to the manufacturer's instructions. Fluorescence intensity is measured at a wavelength of 590 nm using an EnSpire® Multimode Plate Reader multiplate analyzer (PerkinElmer - Italy).

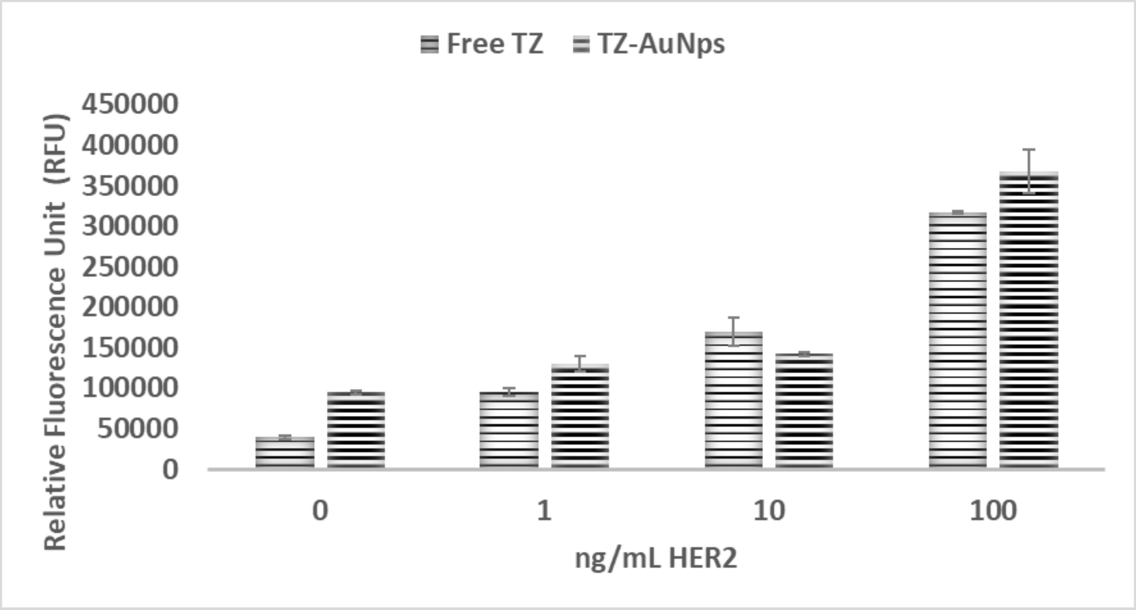

Figure S2 Recognition efficiency of free TZ antibody and TZ-AuNps for HER2 biomarker. The relative fluorescence unit (RFU) is indicated on the y-axis, while the different HER2 concentrations are on the x-axis.

TZ-AuNps SERS performance

To obtain an estimate of the gain experienced by each molecule on each TZ-AuNps, we calculated the enhancement factor (EF) as follows:

$$EF = \frac{I_{SERS}}{I_{Raman}} \frac{N_{Raman}}{N_{SERS}}$$

where I_{SERS} and I_{Raman} are the intensities of the SERS and Raman band at 1080 cm^{-1}, normalized to laser power. N_{Raman} is the number of 4-MBA molecules analyzed in the scattering volume in the Raman experiments and was calculated considering the density of bulk 4-MBA (1.5×10-21 g/nm^3) and its molecular weight (154.19 g/mol). N_{SERS} is the number of molecules bound to the TZ-AuNps surface and probed in the scattering region by the SERS experiments[1]. N_{SERS} was estimated by multiplying the scattering area, the number density of absorbed molecules on the nanospheres

surface (about 4 molecules/nm^2) and the number density of AuNps[2]. The number density of AuNps (2.5×10^{-5} /nm^2) on the slide was determined using atomic force microscopy.

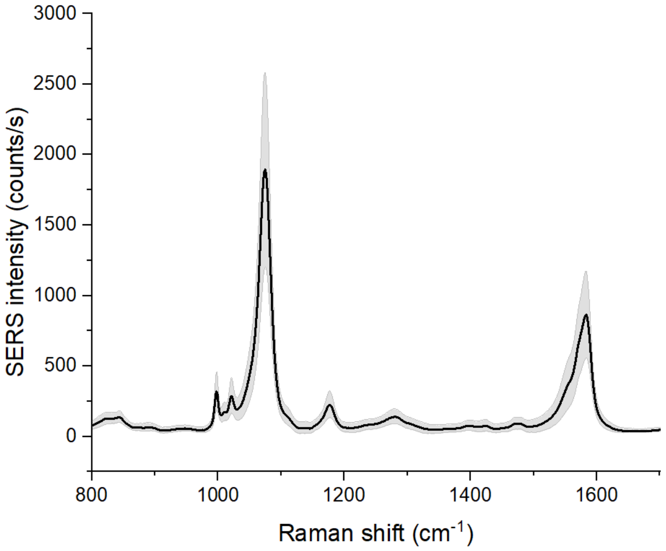

Figure S3 Average spectra of 4-MBA SERS signals.

SERS image elaboration

To highlight the presence of Raman bands associated with the Raman reporter (4-MBA), we show the color maps in terms of the intensity of the bands at 1080 and 1580 cm^{-1}, respectively. To quantify the SERS response, we counted the characteristic spectral peaks of the 4-MBA arising from the background noise and occurring at both the 1080 and 1580 cm^{-1} Raman shifts.

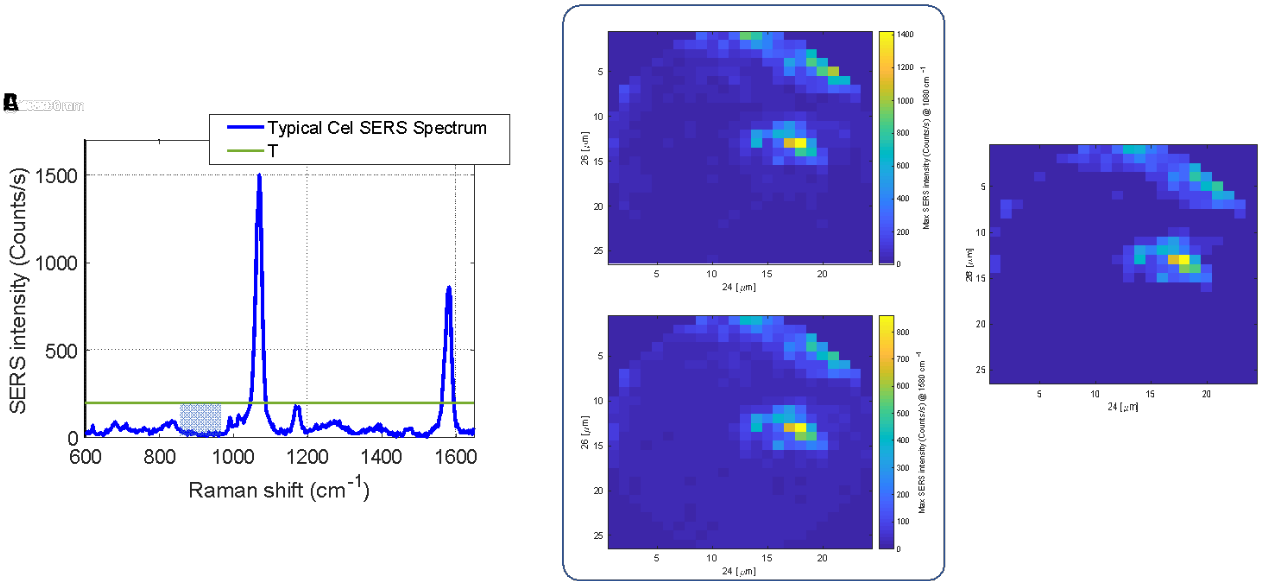

Figure S4 Workflow for determining the “number of pixels” in a SERS map. A) SERS positive control spectra highlighting the threshold set, B) Raw data SERS intensity maps corresponding to 1080 (upper panel) and 1580 cm^{-1} (lower panel) peaks, C) Intensity map considering the number of pixels with intensity above the threshold.

PCA analysis

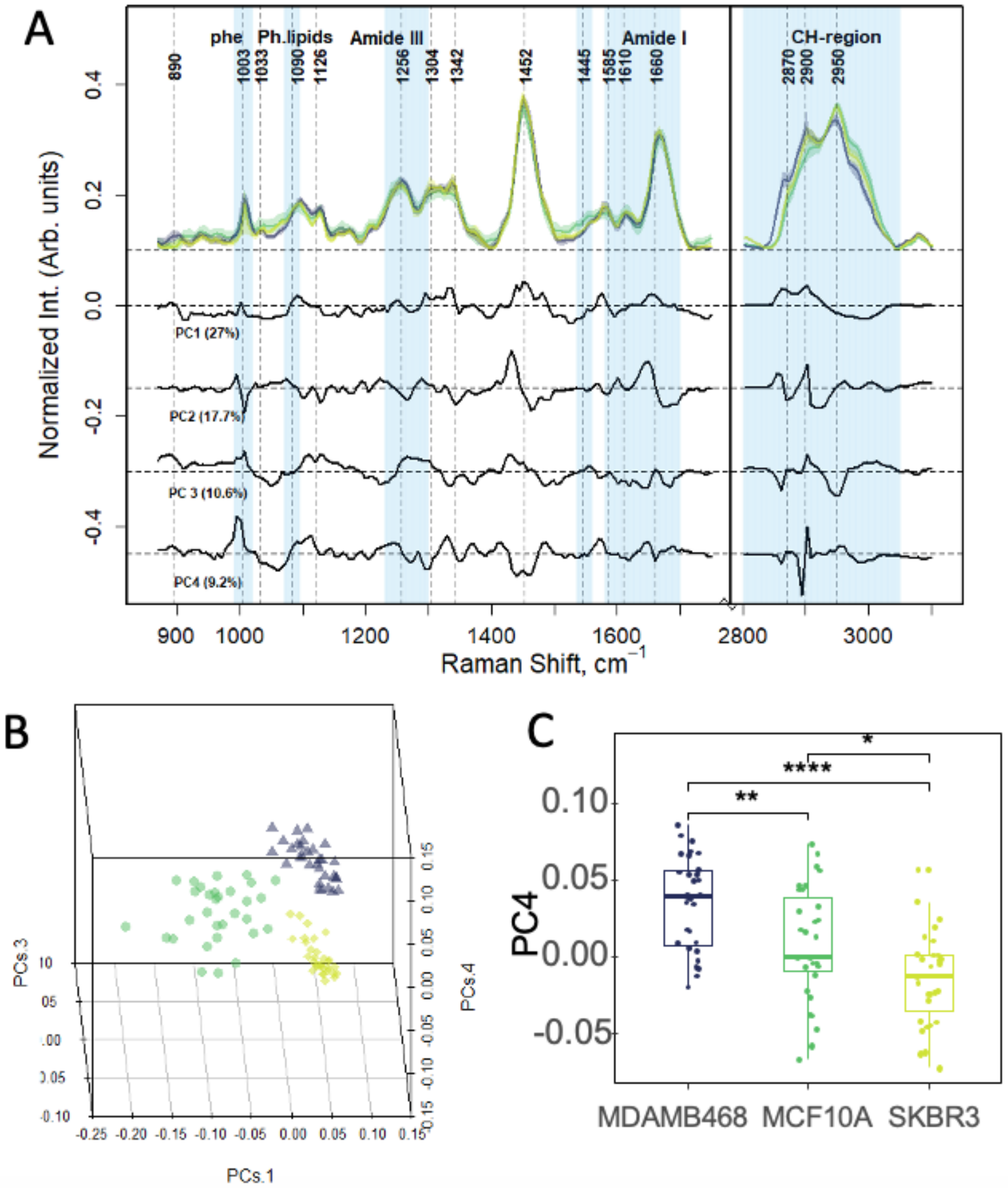

Figure S.5: Principal component analysis of BC Raman spectra on single cells. A, Median IQR Raman spectra of SKBR3 (lime), MFC10A (green), MDAMB468 (dark blue) and the PC1-4 loadings, highlighting the spectral meaningful features for the classification; B, 3D plot of PC1-3-4 score showing the best separation among the BC cell lines. C, boxplot with statistical (Wilcoxon test) difference on PC4 scores, for the different BC Cell lines.

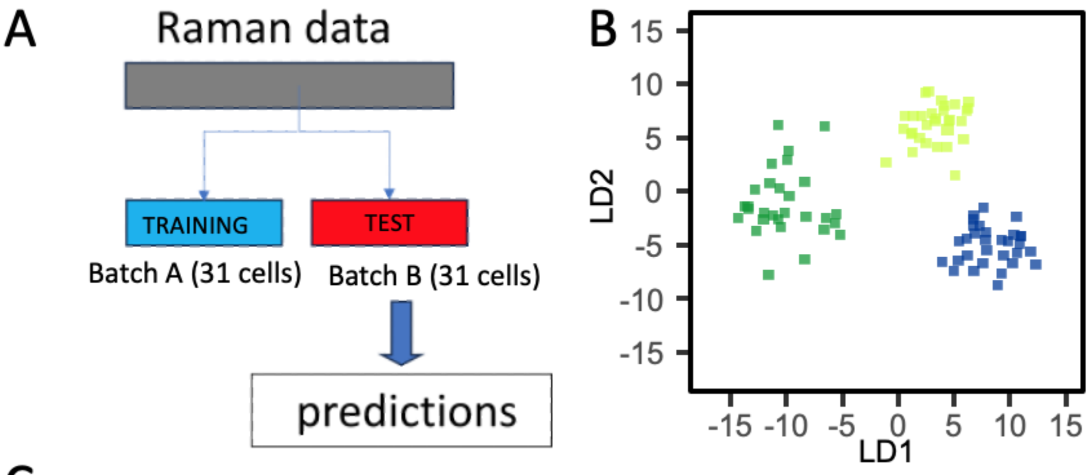

C

Tested/ Predicted	MCFA10A	MDA-MB-468	SKBR3
MCFA10A	31	0	0
MDA-MB-468	0	31	0
SKBR3	0	0	31

Figure S.6: LDA model evaluation and output. A scheme with training and set composition, on two independent batches of cells (31 spectra per type, one spectrum per cell); B, LD1-LD2 latent variables plot, from single cell analysis, and independent test batch of cells. C confusion matrix from LDA model.

References

1. Managò, S., et al., *Bioderived three-dimensional hierarchical nanostructures as efficient surface-enhanced raman scattering substrates for cell membrane probing.* ACS Applied Materials & Interfaces, 2018. **10**(15): p. 12406-12416.
2. Ghalla, H., et al., *Intermolecular interactions and molecular docking investigations on 4-methoxybenzaldehyde.* 2018. **149**: p. 291-300.
