# Supplementary material for: Combined SERS-Raman screening of HER2-overexpressing or silenced breast cancer cell lines
Source: J Nanobiotechnology. 2024 Jun 20;22:350. doi: 10.1186/s12951-024-02600-7 (PMC11188264; doi:10.1186/s12951-024-02600-7)
Supplement: Supplementary file 2 — Supplementary Material 2 [file 12951_2024_2600_MOESM2_ESM.pdf]

Additional file 1: supplementary material as PDF (.pdf) file, including the following sections: materials and reagent; **table S1** Trasduzumab-Nanoparticles (Tz-NPs) characterization by DLS (dynamic light scattering); **figure S1** Particle sizing using DLS. **figure S2** Tz-NPs recognition efficiency by ELISA of naked and conjugated antibody; **figure S3**, averaged SERS signal of 4-MBA Raman reporter; **figure S4** workflow for SERS imaging elaboration. **figure S5**, PCA analysis. **figure S6**, LDA validation.
